# A Novel Design of Spike-Shaped Miniaturized 4 × 4 MIMO Antenna for Wireless UWB Network Applications Using Characteristic Mode Analysis

**DOI:** 10.3390/mi14030612

**Published:** 2023-03-07

**Authors:** Ankireddy Chandra Suresh, Thatiparthi Sreenivasulu Reddy, Boddapati Taraka Phani Madhav, Samah Alshathri, Walid El-Shafai, Sudipta Das, Vishal Sorathiya

**Affiliations:** 1 Department of Electronics and Communication Engineering, Sri Venkateswara University College of Engineering, SV University, Tirupathi 517502, Andhra Pradesh, India; 2 Antennas and Liquid Crystals Research Center, Department of Electronics and Communication Engineering, Koneru Lakshmaiah Education Foundation, Guntur 522302, Andhra Pradesh, India; 3 Department of Information Technology, College of Computer and Information Sciences, Princess Nourah bint Abdulrahman University, P.O. Box 84428, Riyadh 11671, Saudi Arabia; 4 Security Engineering Lab, Computer Science Department, Prince Sultan University, Riyadh 11586, Saudi Arabia; 5 Department of Electronics and Electrical Communications Engineering, Faculty of Electronic Engineering, Menoufia University, Menouf 32952, Egypt; 6 Department of Electronics and Communication Engineering, IMPS College of Engineering and Technology, Malda 732103, West Bengal, India; 7 Faculty of Engineering and Technology, Parul Institute of Engineering and Technology, Parul University, Waghodia Road, Vadodara 391760, Gujarat, India

**Keywords:** characteristic mode analysis, defected ground system, envelop correlation coefficient, isolation, MIMO, UWB, X-band

## Abstract

In this article, a 4 × 4 miniaturized UWB-MIMO antenna with reduced isolation is designed and analyzed using a unique methodology known as characteristic mode analysis. To minimize the antenna’s physical size and to improve the isolation, an arrangement of four symmetrical radiating elements is positioned orthogonally. The antenna dimension is 40 mm × 40 mm (0.42λ_0_ × 0.42λ_0_) (λ_0_ is the wavelength at first lower frequency), which is printed on FR-4 material with a width of 1.6 mm and $\varepsilon_{r}\;$= 4.3. A square-shaped defected ground framework was placed on the ground to improve the isolation. Etching square-shaped slots on the ground plane achieved the return losses S_11_ < −10 dB and isolation 26 dB in the entire operating band 3.2 GHz–12.44 GHz (UWB (3.1–10.6 GHz) and X-band (8 GHz–12 GHz) spectrum and achieved good isolation bandwidth of 118.15%. The outcomes of estimated and observed values are examined for MIMO inclusion factors such as DG, ECC, CCL, and MEG. The antenna’s performances, including radiation efficiency and gain, are remarkable for this antenna design. The designed antenna is successfully tested in a cutting-edge laboratory. The measured outcomes are quite similar to the modeled outcomes. This antenna is ideal for WLAN and Wi-Max applications.

## 1. Introduction

It is desirable to employ this modern technology, known as Ultra-wideband communication devices, to address the needs of high data rates at low costs. Since the Federal Communications Commission (FCC) approved the unlicensed 3.1 GHz–10.6 GHz range for UWB applications; it has evolved into a well-known innovation in the wireless communication industry. Because they constitute an essential part of UWB communication systems, UWB antennas have consequently generated enormous scholarly and scientific attention in recent years [1]. A technological breakthrough known as UWB allows for reliable wireless connectivity with greater capacity and data speeds. However, UWB has limited short-range characteristics as a result of how low power is handled. Due to congestion issues with fading, multipath mitigation, and low power handling capabilities, UWB is thus limited to residential applications [2]. To overcome the issues mentioned above, the MIMO technique was combined with UWB technology. Higher bandwidth and data rates are possible with MIMO technology while raising total sent power. By using proper and appropriate antenna elements at both ends of the communication system, the maximum energy efficiency of the communication system can be achieved without affecting the power capacities of the communication system. Given that we can simply replace the MIMO parts while the transmitted power stays constant, we should arrange the appropriate MIMO antennas to increase channel capacity [3].

Introducing more components causes mutual coupling to rise, which lowers MIMO’s performance. Since electromagnetic interaction increases when MIMO units are put closely together and lowers MIMO performance, low mutual coupling is a crucial MIMO component. These techniques are used to initiate reducing mutual coupling: (1) an improper ground structure, (2) networks for decoupling, (3) parasitic components, (4) electromagnetic band gap (EBG), (5) lines of neutralization, and (6) meta material. To fulfill the expectations of the Internet of Things for more bandwidth and data rates, researchers have started to create UWB antennas to attain a high level of isolation in MIMO Antennas [4]. Two circular patches that are orthogonal to one another constitute the antenna. Two notched bands can be generated by combining a y-shaped slot, a rectangular slot, and a circumferential slot [5]. Innovative approaches are developed for enhancing isolation, bandwidth, and gain. Regarding IoT applications, bidirectional UWB over a multi-mode link increased data rates to 2 Gbps at a lesser cost [6]. To increase impedance matching and achieve 20 dB isolation, the boundaries of triangular-shaped loadings are embedded with a modified Koch fractal structure in MIMO antennas [7]. The Circular UWB-MIMO antenna demonstrated impressive isolation of 28 dB and IBW 134.68% by utilizing a T-shaped slot and protruding strips [8]. Such components allowed the planned antenna to function between 24.1 GHz and 27.18 GHz and between 33 GHz and 44.13 GHz [9]. A 2 × 2 double band antenna composed of the optically reflecting surface AgHT 8 has been planned for use in Wireless LAN and Area network operations [10]. A 4 × 4 planer UWB was created employing circular monopoles as radiators, and isolation was enhanced using DGS and EBG structures. This modified EBG, mushroom shape produced the isolation of more than 17.5 dB across the whole frequency range of 3.0 to 16.2 GHz [11].

The 4 × 4 small antennas have 25 mm × 50 mm of surface area, and PIN diodes were utilized to turn on each radiator in the set up. An LC-shaped decoupling stub was employed for high isolation (2–12 GHz) across the band [12]. A 4 × 4 spike Ultra-wideband antenna with a 50 mm × 25 mm footprint uses dropouts to block Wireless LAN bands between 4.9 GHz and 6.4 GHz. This antenna maintains good isolation from 2 GHz to 12 GHz. All four components for polarization diversity are in opposition to one another. An LC decoupling stub was employed to increase isolation and decrease ECC [13]. A tiny circular-shaped 4 × 4 UWB-MIMO has the following measurements: 44 × 44 × 1.6 mm^3^, and each radiator has a U-shaped slot. Excellent isolation and diversity measurements were attained [14] as a result of the crescent slot on the circular radiator and the circular slot resonator. Using the 4 × 4 rectangle MIMO antenna with an arrow-shaped etching on the rectangular patch, the isolation was increased by more than 18 dB [15]. A two-port UWB-MIMO antenna with semi-circular radiating elements was able to achieve 55 dB isolation and a 37 GHz bandwidth by employing a decoupling stub on the ground. The antenna is 18 mm × 36 mm × 1.6 mm, and it was created utilizing the space diversity technique to boost impedance and bandwidth [16]. A new technique was used to create a multi-input multi-output (MIMO) 4 × 4 elliptical monopoles Ultra-Wide-band (UWB) device with a tiny footprint (45 mm × 45 mm × 1.6 mm). An H-shaped slot and a C-shaped slot were combined in this design to provide double band rejection performance at 5.5 GHz and 7.5 GHz. A stub was connected to the edge of each defective ground structure to provide isolation of 22 dB [17]. A compact 4 × 4 octagonal Koch fractal shape was employed to attain a modest size. Four radiators were positioned diagonally in the 2 GHz–10.6 GHz frequency range, and grounded stubs were employed to increase isolation by 17 dB [18]. To achieve greater isolation and good radiation performance metrics, researchers applied techniques such as orthogonal, Asymmetric Coplanar Strip (ACS), polarization diversity, space diversity, slotted annular ring, and decoupling stubs [19,20,21,22,23,24,25].

But the above methods are not able to enhance the isolation. Therefore, we need an advanced antenna design approach that offers us great isolation and good diversity performance. A method of evaluation that is step-by-step is characteristic mode analysis. This design method is used to analyze antenna properties without using feed. The antenna structure is physically examined using CMA.

The following sections compose the remaining document: In Section 2, the size and design of the antenna are explained. In Section 3, the proposed antenna is tested with the characteristic mode analysis. The simulated and observed findings are discussed in Section 4. In Section 5, the newly created antenna is contrasted with current models. Section 6 concludes the essay.

## 2. Design of 4 × 4 Spike-Shaped UWB Antenna

The designed antenna radiator is in the form of a spike-shape. The four symmetrical monopoles are placed orthogonal to one other, resulting polarization diversity. The proposed spike-shaped MIMO antenna has size in the order of 40 mm × 40 mm × 1.6 mm (0.44λ_0_ × 0.44λ_0_ × 0.0176λ_0_), placed on a Fr-4 material with loss tangent of 0.002 and permittivity of 4.3. The Computer Simulation Technology (CST) is used to design and simulate the proposed antenna.

The spike-shaped single radiator is a circular patch with a radius of 4 mm and it has five spike bubbles with a diameter of 1.5 mm placed on its circumference. The feeder line has a width of 1.2 mm, length is of about 10.2 mm, and the distance between two radiators is 6.25 mm. In this design, a novel ground structure shape is used to achieve good radiation performance and better isolation.

**Figure 1:**
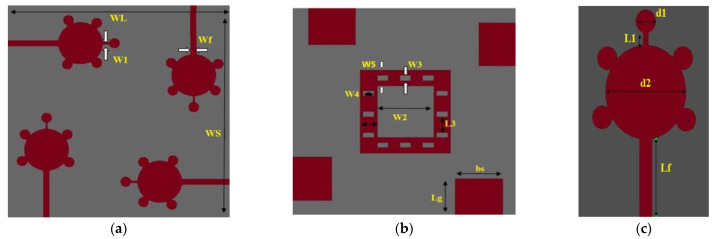
Orientation of the designed antenna (**a**) top view, (**b**) bottom view, (**c**) single element.

All four radiators are of the same dimensions and are arranged in an orthogonal pattern. By using this arrangement of elements in MIMO, it is possible to reduce the mutual coupling among patch elements. Four rectangular-shaped patches with a width of 8.334 mm and a length of 7 mm form the structure of the ground plane. In order to achieve good isolation, a square-shaped conducting patch with a width of 3 mm is placed on the ground at a distance of 5 mm from the center of ground plane, as shown in Figure 1b. To improve the bandwidth and isolation in UWB and ITU bands, 12 rectangular slots are placed on 3 mm square ground patch.

Out of those 12 rectangular slots, 6 slots are placed vertically, and the remaining slots are placed horizontally on a square-shaped ground patch as shown in Figure 1b. The dimensions of a 4 × 4 spike-shaped Ultra-wide band antenna are shown in Table 1.

### Theory of Characteristic Mode Analysis

Characteristic mode theory is used to analyze the antenna’s input impedance and current distributions without any excitation. The characteristic mode theory (CMT) is used to study radiation patterns and scattering fields in perfect electric conductors. In perfect electric conductors, the antenna’s input impedance and radiation pattern are proportional to the total surface current density at the feeding point [26]. The impedance matrix is represented in the below equation.

(1) $$\;\;\;\;z_{imp}=R_{real}+jI_{img}$$

(2) $$\left[I\right]\vec{j_{n}}=\lambda_{n}\left[R\right]\;\vec{j_{n}}$$

where R is the real part and I is the imaginary part of impedance matrix. Here λ_n_ denotes the Eigen Vector’s eigenvalues. J_n_ is the antenna current defined in terms of its characteristic modes

(3) $$\vec{j_{n}\;\;}\;=\sum_{n}^{N}\frac{\vec{J_{n\;}}\vec{E^{i}}}{1+j\lambda_{n}}\vec{J_{n}}=\sum_{n}^{N}\alpha_{n}\vec{J_{n}}\;$$

where N is the order of the moment matrix and J_n_ is CM current, $\lambda_{n}$ eigen values and $E^{i}$ is incident electric field. Equation (1) explains the characteristic modes in the antenna structure. Equation (1) has two terms $\vec{J_{n}}\vec{E^{i}}$ and $\frac{1}{\;1+{j\lambda }_{n}}$.

The dot product of $E^{i}$ and J_n_ is zero at all points except the feed point of the conducting antenna. The overall phase of $E^{i}$ depends on eigen values λ_n_.

## 3. Evaluation Procedure of 4 × 4 UWB-MIMO Antenna

The characteristics mode analysis (CMA) is used to develop the proposed antenna. The entire design process is carried out in four design stages performed in the proposed antenna design evaluation process. The total designing process of the suggested antenna depends on CMA metrics such as (1) Eigen values, (2) characteristic angle, and (3) modal significance. These three properties exist in each characteristic mode. In this current antenna design, only the modal significance parameter was considered. The Spike-shaped 4 × 4 MIMO antenna is analyzed and investigated in four stages: antenna0 (Ant#0), antenna1 (Ant#1), antenna2 (Ant#2), and antenna3 (Ant#3) [27]. The CM currents in CMA are used to evaluate the performance of each characteristic mode. Without using any excitation, these characteristic mode current distributions can be observed. The proposed antenna achieved a good impedance bandwidth (IBW) covering the bandwidth requirements of UWB systems and also X-band.

**Figure 2:**
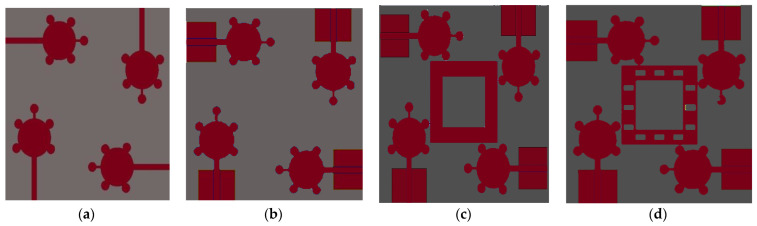
Step by step design procedure of spike antenna. (**a**) Ant#0 (**b**) Ant#1 (**c**) Ant#2 (**d**) Ant#3.

The proposed antenna was developed using a CMA method. A series of sequential procedures are used throughout the designing process. The modal current distribution in Computer Simulation Technology (CST) is demonstrated using multi-layer solver. The evaluation process of antenna in step-by-step manner is depicted in Figure 2

**Figure 3:**
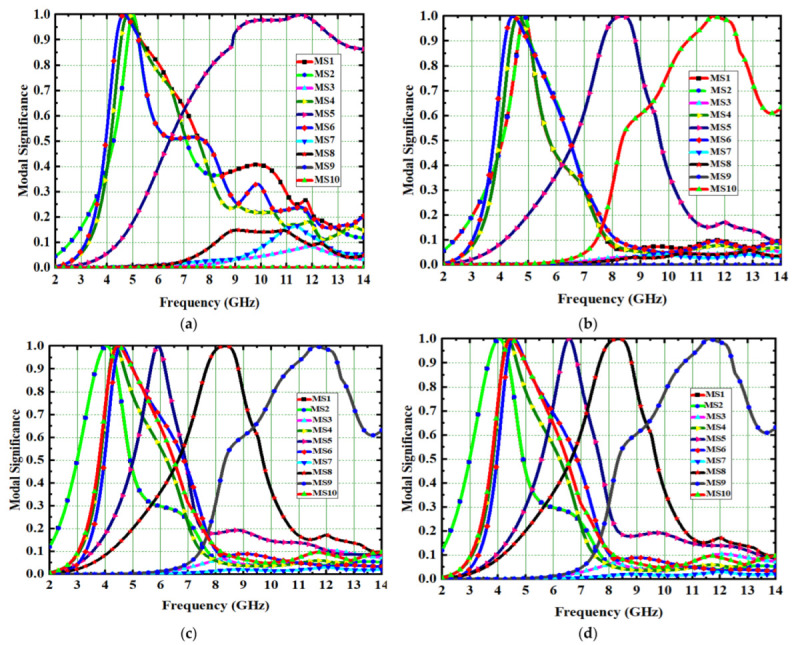
Spike antenna model significances. (**a**) Ant#0, (**b**) Ant#1, (**c**) Ant#2, (**d**) Ant#3.

Modal significance is used to implement the proposed antenna in CMA design. Without using any excitation signal, the entire antenna design process is carried out in four evaluation stages from Ant#0 to Ant#3. The Ant#0 is made up of four spike-shaped radiators that are printed on FR-4 substrate. Each spike radiator has a radius of 4 mm with spike bubble radius of 1 mm. The Ant#0 has no conducting ground plane and is able to generate ten characteristic modes using the multilayer solver. Only five out of ten characteristic modes (CMs) contribute bandwidth and isolation. The modal significances of Ant#0 are shown in Figure 3a. The characteristics modes CM1, CM2, CM4, CM5, and CM6 contribute bandwidth between 4 GHz and 6 GHz. The remaining modes CM3, CM7, CM8, CM9, and CM10 do not contribute any bandwidth. The Ant#0 is good at mid-band frequencies (4 GHz–6 GHz) and not good at low and high frequencies. In Ant#0, the absence of a ground plane produces high mutual coupling at low and high frequencies. Ant#0′s design was changed to improve isolation and bandwidth. On the ground plane, rectangular ground edges were added for each radiator. The rectangular ground edges are 8.33 mm and 7 mm in width and length, respectively. Hence, Ant#0 becomes Ant#1 after the ground edges are added. Ant#1′s characteristic modes were examined in the absence of excitation. Ant#1 generates ten different modes. The majority of the characteristics modes, CM1, CM2, CM4, and CM6, are concentrated at mid frequencies (4 GHz–8 GHz) while CM5 and CM10 are concentrated in between 8 GHz and 11 GHz. The remaining modes, CM3, CM7, CM8, and CM9, are ineffective and contribute no bandwidth and isolation, this is due to the addition of ground edges in the ground plane. Figure 3 depicts the corresponding modal significances on the ground plane, a 3 mm square-shaped conducting patch is added to convert Ant#1 to Ant#2 and analyze its characteristic modes. Some CMs are below 4.5 GHz, some are at mid-band frequencies, and some are at 11 GHz, implying that this antenna achieved good bandwidth but requires more isolation. In Figure 3c, the corresponding modal significances of Ant#2 are shown. Ant#2 was transformed into Ant#3 by adding 12 rectangular slots on 3 mm square patch in ground plane. Ant#3 can be examined in terms of its characteristic modes. The CM3 and CM7 does not contribute any bandwidth in the ten modes.

**Figure 4:**
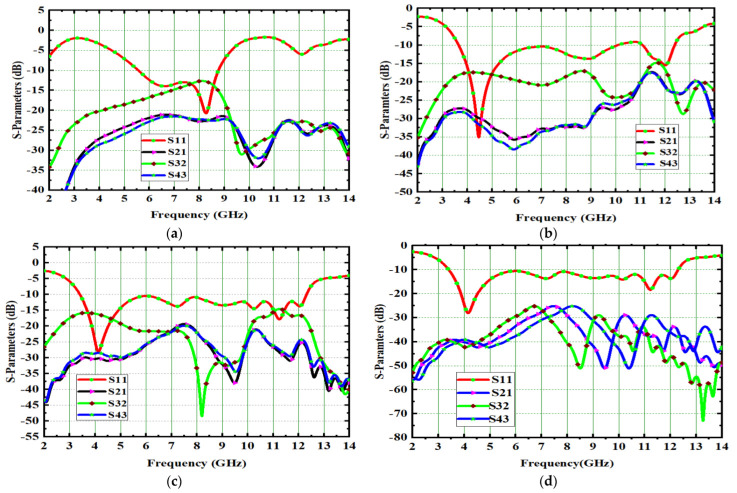
Spike antenna S-parameters. (**a**) Ant#0, (**b**) Ant#1, (**c**) Ant#2, (**d**) Ant#3.

The remaining modes, CM1, CM2, CM4, CM5, CM6, CM8, CM9, and CM10, not only covers UWB but also the X-band (8 GHz–12 GHz). Figure 3d shows the corresponding modal significances. The CM4 is resonated at 4.1 GHz during the evaluation process from Ant#0 to Ant#3. As a result, CM4 is referred to as the dominant mode. All CMs are scattered in between 3 GHz and 12 GHz except CM3 and CM7. The feed is applied to all antennas and their corresponding S-Parameters are depicted in Figure 4. Placing the square-shaped ground structure in the proposed antenna, the current interactions among the radiators reduce because of the increase in current interaction between the ground conduction patch and radiators.

**Figure 5:**
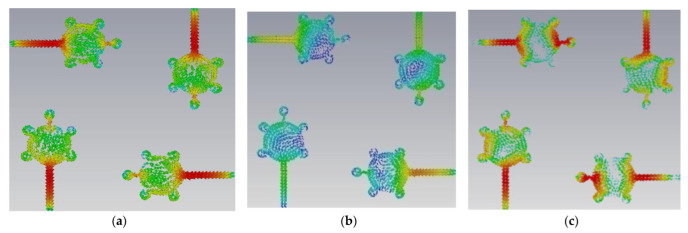
Current distribution effects on spike antenna at different frequencies in CM4 at (**a**) 4.1 GHz, (**b**) 7.2 GHz, (**c**) 10.1 GHz.

**Figure 6:**
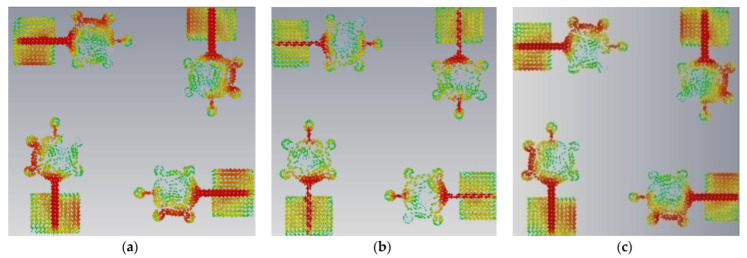
Current distribution effects on spike antenna at different frequencies for CM4 in Ant#0 at (**a**) 4.1 GHz, (**b**) 7.2 GHz, (**c**) 10.1 GHz.

**Figure 7:**
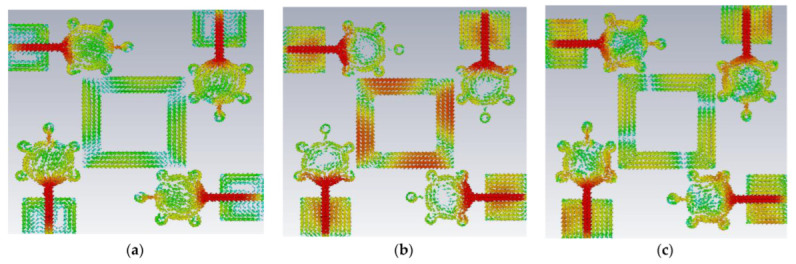
Current distribution effects on spike antenna at different frequencies for CM4 in Ant#1 at (**a**) 4.1 GHz, (**b**) 7.2 GHz, (**c**) 10.1 GHz.

**Figure 8:**
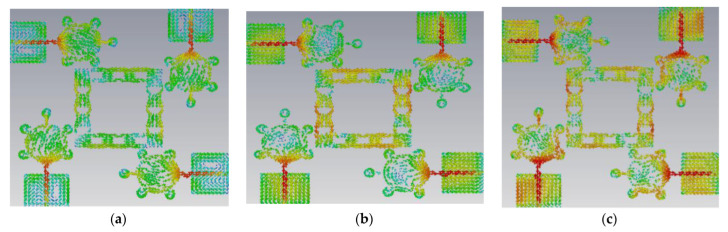
Current distribution effects on spike antenna at different frequencies for CM4 in Ant# 2 at (**a**) 4.1 GHz, (**b**) 7.2 GHz, (**c**) 10.1 GHz.

**Figure 9:**
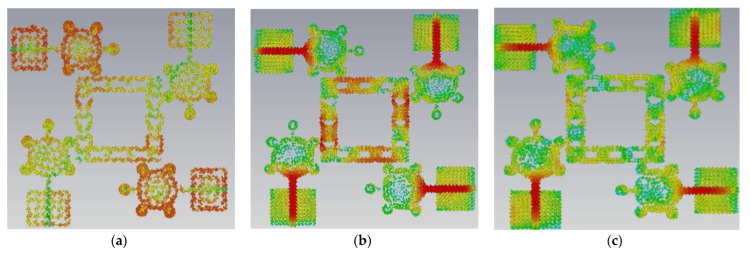
Current distribution effects on spike antenna at different frequencies for CM4 in Ant#3 at (**a**) 4.1 GHz, (**b**) 7.2 GHz, (**c**) 10.1 GHz.

This antenna is suitable for UWB (3.1–10.6 GHz) and X-band applications (8–12 GHz). Figure 5, Figure 6, Figure 7 and Figure 8 shows the characteristic current distributions of antennas Ant#0 to Ant#3 at 4.1 GHz, 7.2 GHz, and 10.1 GHz. Figure 9 shows Ant#3′s current distributions at various frequencies.

## 4. Results of 4 × 4 Spike-Shaped UWB-MIMO Antenna

**Figure 10:**
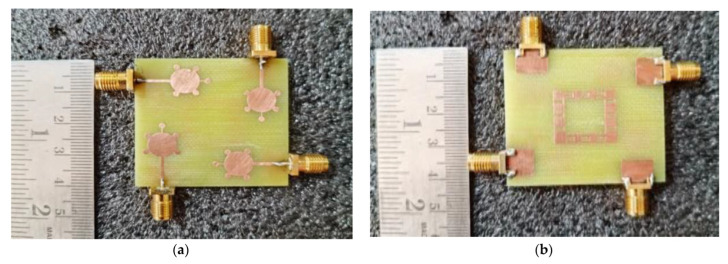
Fabricated prototype model of proposed spike antenna (**a**) front view, (**b**) back view.

**Figure 11:**
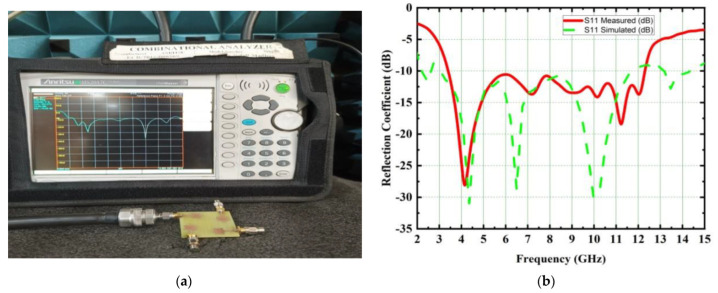
Simulated and measured reflection coefficients of the proposed spike antenna (**a**) S_11_ measurement set up using VNA, (**b**) simulated and measured S_11_ comparison.

**Figure 12:**
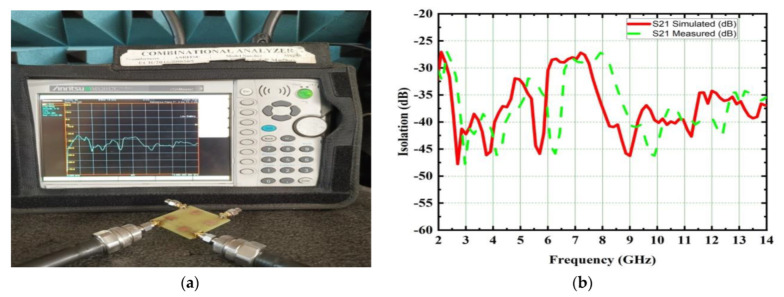
Simulated and measured isolation characteristics of the proposed spike antenna (**a**) S_21_ measurement set up using VNA (**b**) simulated and measured S_21_ comparison.

The proposed UWB-MIMO spike-shaped antenna has good MIMO metrics such as radiation characteristics and isolation. The CMA technique is used to design the spike-shaped antenna, resulting in improved isolation. From 3.2 GHz to 12.44 GHz, the impedance bandwidth is 9.24 GHz and it covers the bandwidth requirements for UWB and ITU bands. Figure 10 depicts the designed antenna prototype. The experimental set up for measuring the reflection coefficient using VNA is shown in Figure 11a. Figure 11b compares both the simulated and measured reflection coefficients (S_11_). The designed antenna provides isolation of 26 dB. This isolation is good in MIMO antenna metrics. The experimental set up for measuring the isolation using VNA is shown in Figure 12a. Figure 12b provides a comparative analysis of simulated and experimental S_21_ values. The novel type of decoupling structure in the ground plane provides the excellent isolation.

**Figure 13:**
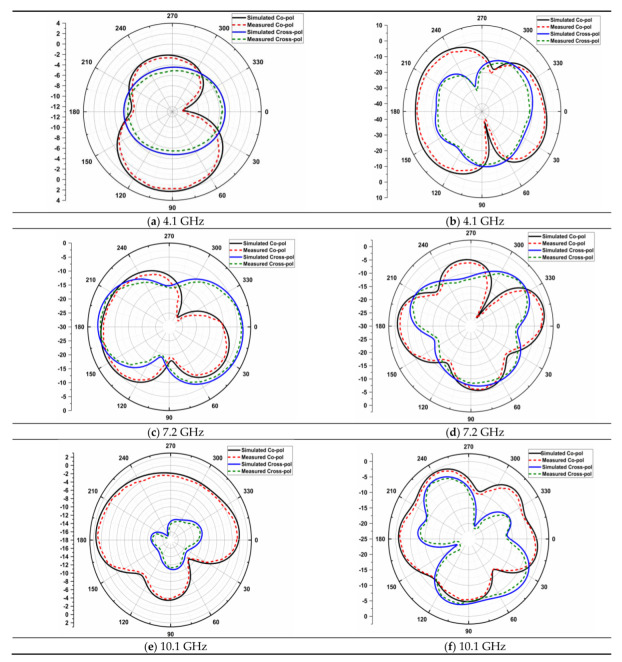
Radiation patterns of spike antenna at various frequencies in E (**a**,**c**,**e**) and H (**b**,**d**,**f**)—planes.

**Figure 14:**
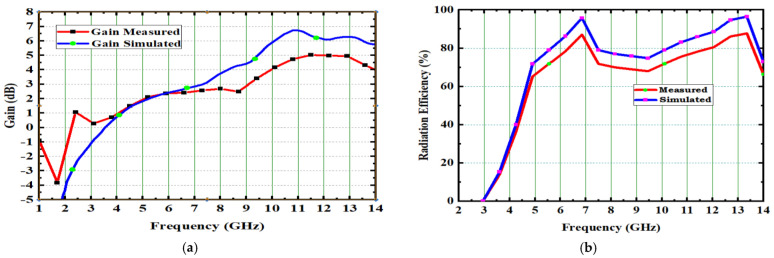
Gain and radiation efficiency of spike antenna. (**a**) Gain vs. Frequency. (**b**) Radiation Efficiency vs. Frequency.

Figure 13 shows the 4 × 4 radiation patterns at 4.1 GHz, 7.2 GHz, and 10.1 GHz. The E-plane and H-plane of the primary radiator are obtained by activating its port and other ports are connected with 50 ohm load. An anechoic chamber set up is used to measure the radiation pattern, with the aid of the DRH20. The radiation efficiency is achieved as 89% and gain is 4.9 dB as shown in Figure 14.

### 4.1. Spike-Shaped UWB-MIMO Antenna Performance

**Figure 15:**
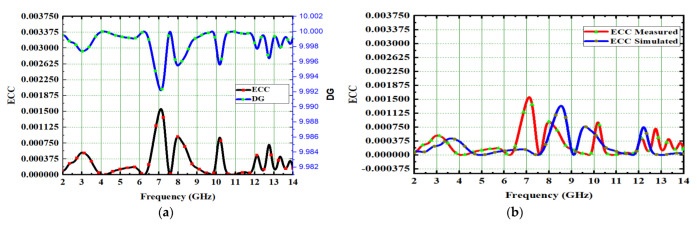
Spike antenna (**a**) DG and ECC, (**b**) ECC measured and simulated.

Four performance metrics exist in MIMO antenna systems, all of which should be acceptable [28,29,30]. The envelope correlation coefficient is among the key diversity factors for assessing MIMO performance. The ECC describes the interactions between the MIMO elements, it should be ideally zero. Over this operating band, a value of less than 0.5 is acceptable. S-parameters and far-fields can be used to calculate the ECC. The following equation can be used to represent the ECC [28].

(4) $$\rho_{ij}=\frac{{\left|S_{\mathrm{kk}}{}^{*\;}S_{\mathrm{km}}+S_{\mathrm{mk}}{}^{*}S_{\mathrm{mm}}\right|}^{2}}{\left(1-\left({\left|S_{\mathrm{kk}}\right|}^{2}+{\left|S_{\mathrm{mk}}\right|}^{2}\right)\right)\left(1-\left({\left|S_{\mathrm{mm}}\right|}^{2}+{\left|S_{\mathrm{km}}\right|}^{2}\right)\right)}$$

where k = 1 and m = 2. Using sophisticated decoupling methods and characteristic mode analysis this antenna achieved ECC of 0.0016 and is depicted in Figure 15b. The lesser ECC value means the antenna elements were well-isolated in MIMO. The envelope correlation coefficient (ECC) is also computed from far fields [29] and shown in Equation (5).

(5) $$P_{e}=\frac{{\left|\int_{0}^{2\pi }\int_{0}^{\pi }\left(XPR.E_{\theta 1.}E_{\theta 2}^{*}P_{\theta }+E_{\Phi 1}E_{\Phi 2}^{*}.\;P_{\Phi }\right)d\Omega \;\;\;\;\right|}^{2}}{\int_{0}^{2\pi }\int_{0}^{\pi }\left(XPR.E_{\theta 1.}E_{\theta 1}^{*}P_{\theta }+E_{\Phi 1}E_{\Phi 1}^{*}.\;P_{\Phi }\right)d\Omega X\int_{0}^{2\pi }\int_{0}^{\pi }\left(XPR.E_{\theta 2.}E_{\theta 2}^{*}P_{\theta }+E_{\Phi 2}E_{\Phi 2}^{*}.\;P_{\Phi }\right)d\Omega }$$

Another important MIMO diversity parameter is diversity gain (DG). Generally, in MIMO antennas the DG is nearly 10 dB. Formula [28] illustrates how such DG is stated in regards of ECC.

(6) $$\mathrm{DG}=10\sqrt{1-ECC^{2}}$$

Figure 15a shows that the DG is nearly 9.962 dB. The channel capacity loss (CCL) is another important diversity parameter [30]. The number of elements in a MIMO system determines its channel capacity. In the equation, the CCL [31] is expressed as:

(7) $$\mathrm{CCL}=-log_{2}\det\left(\psi R\right)$$

$$\psi^{R}=\left[\begin{array}{cccc} \rho_{11} & \rho_{12} & \rho_{13} & \rho_{14}\\ \rho_{21} & \rho_{22} & \rho_{23} & \rho_{24}\\ \rho_{31} & \rho_{32} & \rho_{33} & \rho_{34}\\ \rho_{41} & \rho_{42} & \rho_{43} & \rho_{44} \end{array}\right]$$

$$\rho_{ii}=1-\left({\left|S_{ii}\right|}^{2}+{\left|S_{iJ}\right|}^{2}\right)=-\left|S_{ii}{}^{*}S_{ij\;}+S_{ji}{}^{*}S_{ij}\right|,\;\mathrm{for}i,\;j=1\mathrm{to}4$$

**Figure 16:**
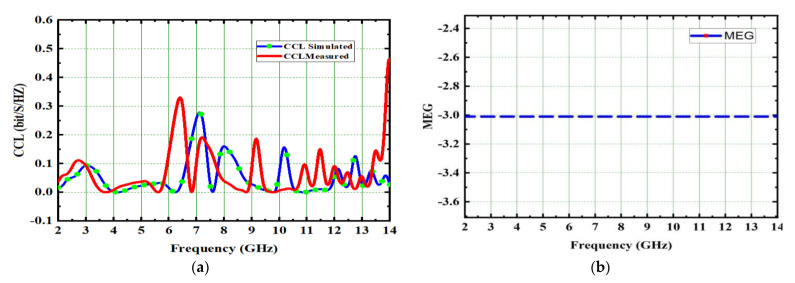
Spike antenna (**a**) CCL, (**b**) MEG.

The acceptable value of CCL is 0.4 bits/s/Hz. In this proposed design, CCL of 0.31 bits/s/Hz is achieved and depicted in Figure 16a.

### 4.2. Mean Effective Gain (MEG)

The MEG can be expressed using Equation (8) [32]

(8) $${\mathrm{MEG}}_{i}=\int_{0}^{2\pi }\int_{0}^{\pi }[\frac{ѓ}{1+ѓ}G_{\theta }\left(\theta ,\Phi \right)P_{\theta }\left(\theta ,\Phi \right)+\frac{ѓ}{1+ѓ}G_{\Phi }\left(\theta ,\Phi \right)P_{\Phi }\left(\theta ,\Phi \right)]sin\theta d\theta d\Phi$$

where ѓ is the event field’s trans discrimination (XPD). The gain components are represented as $G_{\theta }$ and $G_{\emptyset }$ for i^th^ elements. The MEG of the intended antenna is −3.1 dB, as illustrated in Figure 16b.

## 5. Comparison with Existing Models

The spike-shaped 4 × 4 UWB-MIMO antenna is designed to work in both UWB and X-band. This antenna has some better features than others previously reported in the literature [11,15,21,24,25,31,32] and illustrated in Table 2. Compared with other conventional models the designed antenna has the added benefit of the CMA process. In the designed UWB-MIMO antenna, the ECC, IBW, and diversity performances are found to be good.

## 6. Conclusions

The spike-shaped UWB-MIMO antenna operates in the 3.2 GHz–12.44 GHz frequency range, which includes the entire UWB (3.1 GHz–10.6 GHz) and X-band (8 GHz–12 GHz). Four spike-shaped circular patch radiators are placed orthogonally to achieve polarization diversity. The reflection coefficients (S_11_) are below −10 dB, and isolation between both the radiating elements are far better than 26 dB. The radiation parameters include 89% radiation efficiency, 118.15% impedance bandwidth, and a 4.9 dB gain. ECC, DG, MEG, and CCL have diversity features of 0.0016, 9.962 dB, −3.1 dB, and 0.31 bits/s/Hz, accordingly. In a cutting-edge experiment, these findings are verified. According to its performance parameters, the developed antenna is suitable for wireless communication in the spectrum of UWB and X-band.

## Figures and Tables

**Table 1:** Dimensions of spike-shaped 4 × 4 UWB-MIMO.

Parameter	Symbol	Value (mm)	Parameter	Symbol	Value (mm)
Substrate length	W_L_	40	Substrate width	W_s_	40
Length of the spike ground	L_1_	2	Ground length	L_g_	8.33
Radiator diameter	d_2_	8	Feed length	L_f_	10.2
Diameter of spike	d_1_	1	Width of the square ground patch	L_1_	3
Feed width	W_f_	1.2	Space between two vertical slots	L_3_	3
Square patch length	W_2_	10	Width of the ground	bs	7
Width of the slot	W_4_	2	Width of square patch	W_5_	3
Height of the slot	W_3_	1			

**Table 2:** Performance comparison of the proposed extended-UWB-MIMO antenna with state-of-the-art antennas.

Ref	Dimensions (mm^3^)	Impedance Bandwidth (GHz)	Isolation (dB)	Gain (dB)	Radiation Efficiency (%)	ECC
[11]	0.55λ × 0.55 × 0.16λ	3–16.2	>17.5	8.4	>80	<0.3
[15]	0.69λ × 0.69λ × 0.00λ	2.6–11	>17.4	3.99	>85.7	<0.004
[21]	0.38λ × 0.38λ × 0.017λ	3.2–11	>15	4	>70	<0.5
[24]	0.41λ × 0.44λ × 0.01λ	3.1–10.6	>20	4	>90	<0.2
[25]	0.55λ × 0.55λ × 0.015λ	2.84–15.88	>16	6.35	>89	<0.07
[31]	0.56 λ × 0.39 λ	3.52–10.08	>22	2.91	----	<0.04
[32]	0.67 λ × 0.67 λ	2.8–13.3	>18	6	------	<0.06
Prop.	0.44λ × 0.44λ × 0.0176λ	3.2–12.44	>26	4.9	>89	<0.0016

## Data Availability

The data presented in this research are available on request from the corresponding author.
